# Volume Stability of Magnesium Slag-Based Building Materials: A Critical Review of Mechanisms and Mitigation Strategies

**DOI:** 10.3390/ma19142986

**Published:** 2026-07-10

**Authors:** Jialin Liu, Yujiang Du, Yan Liu, Junlin Wang, Wei Zhang

**Affiliations:** 1 Hebei Key Laboratory of Structural Safety and Low-Carbon Construction for Rural Buildings, Hebei Agricultural University, Baoding 071001, China; 2 School of Civil Engineering, Tianjin University, Tianjin 300072, China

**Keywords:** magnesium slag, solid waste, collaborative utilization, recycling

## Abstract

Magnesium slag (MS), an important industrial solid waste generated during the magnesium production process, is recognized for its considerable potential to reduce carbon emissions and advance sustainability in the building materials industry. However, its widespread application is severely hindered by volume instability, which is primarily attributed to the delayed hydration expansion of free calcium oxide and free magnesium oxide. In this review, the physical and chemical properties of magnesium slag are systematically summarized, and the underlying mechanisms responsible for the volume instability of MS-based cementitious materials are critically elucidated. Furthermore, targeted strategies for improving volume stability are focused on and evaluated, including the acid treatment of magnesium slag for oxide passivation and the collaborative utilization with complementary solid wastes such as ground granulated blast furnace slag, steel slag, and fly ash. Through this critical synthesis, a framework is established for overcoming the soundness bottleneck, thereby repositioning magnesium slag as a viable and reliable constituent in next-generation sustainable building materials.

## 1. Introduction

The construction industry has long been recognized as a primary consumer of non-renewable natural resources and a dominant contributor to global anthropogenic carbon dioxide emissions [1,2]. Among its constituent activities, the manufacture of Portland cement alone has been estimated to account for approximately 8% of the total global CO_2_ release. This staggering figure has underscored the pressing necessity for the development and deployment of alternative low-carbon binder systems. In response to escalating sustainability imperatives and increasingly stringent environmental regulations, the development of cementitious materials derived from industrial by-products has been accorded considerable research priority. Such alternative binders have been distinguished not only by their substantially reduced environmental footprint but also by their capacity to valorize voluminous waste streams. Through this valorization pathway, land disposal burdens have been mitigated and virgin mineral resources have been effectively conserved [3,4].

Within the portfolio of industrial solid wastes amenable to construction applications, magnesium slag has been identified as a particularly abundant yet critically underutilized resource [5,6]. This residual material has been generated in vast quantities as a by-product of the Pidgeon process, the prevailing metallurgical route for primary magnesium extraction [7]. Notably, over 80% of global magnesium output is concentrated within China, thereby localizing an immense slag disposal challenge within this region. The production of each metric ton of metallic magnesium has been reported to yield between five and seven metric tons of magnesium slag [8]. Consequently, annual generation rates in China alone have been documented to exceed seven million tons [9,10]. However, the comprehensive utilization ratio of magnesium slag has remained persistently and alarmingly low. Prevailing disposal practices have been largely confined to low-value landfilling or rudimentary backfilling operations. Such management approaches have been unequivocally demonstrated to precipitate severe environmental detriments, including extensive land occupation, fugitive dust emissions, and the potential leaching of heavy metal constituents into surrounding ecosystems [11,12]. The imperative to engineer higher-value applications for magnesium slag, particularly its deployment as a principal component in cementitious matrices, has therefore been recognized as a pivotal strategy for enhancing the circular economy profile of the magnesium production sector [13,14].

Notwithstanding its compositional and mineralogical analogies to conventional Portland cement, which have rendered magnesium slag a theoretically viable eco-substitute, the extensive utilization of magnesium slag-based building materials has been persistently impeded by formidable technical obstacles. Foremost among these challenges has been the intrinsic volumetric instability exhibited by such materials during their prolonged service life. This deleterious behavior has been principally ascribed to the delayed hydration expansion induced by the presence of free calcium oxide (f-CaO) and free magnesium oxide (f-MgO) phases within the slag matrix [15,16]. The expansive forces generated by the gradual conversion of these metastable oxides into their respective hydroxides have been documented to provoke severe micro-cracking, macroscopic fracturing, and catastrophic failure of hardened cementitious composites [17]. Such latent deterioration mechanisms have been unequivocally shown to compromise the long-term structural integrity and safety margins of magnesium slag-based building materials. This phenomenon has thus posed an existential barrier to their acceptance in load-bearing and structural engineering contexts [18,19].

Given the pronounced discordance between the promising low-carbon potential of magnesium slag and the severe impediment posed by its deficient volumetric stability, a comprehensive and critical synthesis of underlying mechanisms and remedial interventions has become urgently warranted [20,21]. This review has been undertaken with the explicit objective of systematically elucidating the fundamental physicochemical attributes of magnesium slag that govern its cementitious behavior. Furthermore, the mechanistic origins of volume instability phenomena in magnesium slag-based cementitious systems have been examined in meticulous detail. A structured and critical appraisal of the principal mitigation strategies currently advanced in the scientific literature has been conducted. These strategies have been systematically categorized to encompass both intrinsic modification protocols applied directly to the magnesium slag substrate and synergistic blending approaches wherein magnesium slag is co-activated with other industrial solid waste streams. Such collaborative solid wastes have included, but have not been limited to, granulated blast furnace slag, steel slag, and fly ash. By consolidating dispersed research findings and providing a coherent framework for understanding the nexus between slag chemistry, hydration trajectory, and dimensional stability, this review is intended to furnish a definitive reference for researchers and practitioners engaged in the sustainable transformation of the built environment. It is anticipated that the insights delineated herein will catalyze the formulation of robust, volume-stable magnesium slag-based construction materials [22].

Thereby, the considerable latent value of this waste stream will be unlocked and the transition toward a resource-efficient and decarbonized construction industry will be accelerated.

## 2. The Physical and Chemical Properties of Magnesium Slag

**Figure 1:**
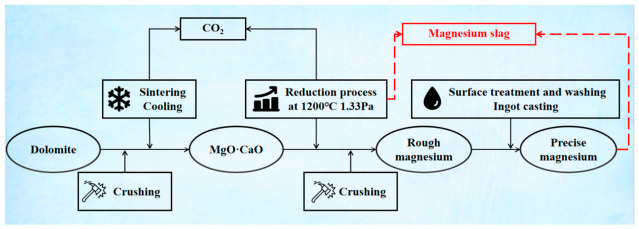
Producing magnesium through the Pidgeon process.

Magnesium slag is a typical calcium–magnesium solid waste produced during the melting of magnesium metal. Unique physical and chemical properties are exhibited by magnesium slag. The preparation of magnesium slag is accomplished by the Pidgeon process (silicon thermal method) and the aluminum thermal method [23,24]. As shown in Figure 1, the melting of magnesium using the Pidgeon process involves the steps of calcination, grinding, reduction, and refining [25,26]. This process is characterized by the advantages of simple operation and high cost-effectiveness; however, the reduction and heat utilization in the melting process are relatively low. In contrast, the aluminum thermal method is regarded as an efficient and cleaner method for magnesium melting. In recent years, it has been considered one of the most advantageous magnesium melting technologies in the context of reducing carbon emissions. Lightweight calcium carbonate and magnesium aluminum spinel are mainly produced by the aluminum thermal method, both of which are of extremely high value. Currently, due to the immaturity of the aluminum thermal method for the magnesium melting process, its widespread application has not yet been realized. The metallurgical slag generated during magnesium metal melting employing the Pidgeon process is focused on in this paper.

**Figure 2:**
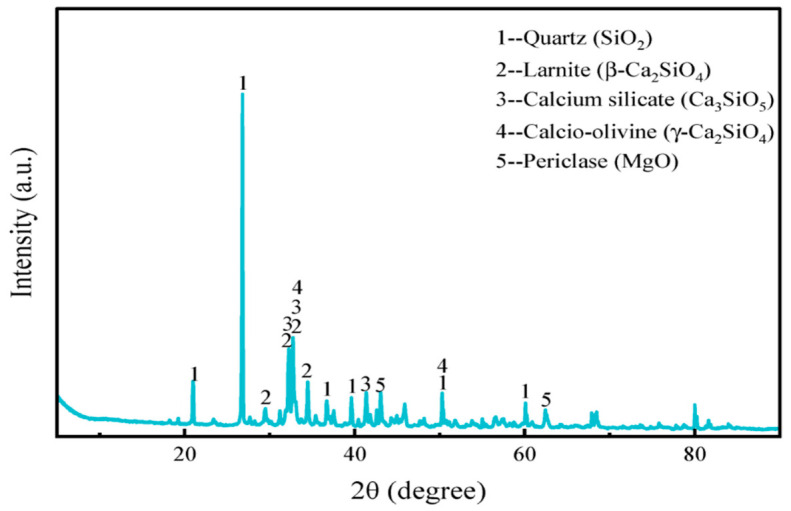
XRD pattern of magnesium slag, adapted from Ref. [30].

The melting point of magnesium slag has been reported to be approximately 1400–1500 °C. Its linear thermal expansion coefficient in the solid state is about 0.0005/°C, and its specific heat capacity is approximately 0.754 J/(g·°C) [27,28]. The true density of magnesium slag generally ranges from 2.86 to 2.92 g/cm^3^, while its loose bulk density is approximately 1.15 ± 0.05 g/cm^3^ [29]. The typical chemical composition of magnesium slag varies within the following ranges: CaO: approximately 55%; SiO_2_: approximately 30%; Al_2_O_3_: approximately 1%; MgO: approximately 12%; and Fe_2_O_3_ approximately 3% [30]. The corresponding XRD patterns are shown in Figure 2 [30]. The dominant mineral phase in magnesium slag is C_2_S, accompanied by small amounts of f-CaO and MgO. During industrial production, magnesium slag is subjected to different cooling processes, which may alter the crystal structure of C_2_S and consequently affect the chemical reactivity of the slag. To investigate the influence of cooling conditions on the properties of magnesium slag, three cooling regimes have been simulated under laboratory conditions: natural cooling, forced-air cooling, and water quenching. Under natural cooling, the relatively slow cooling rate promotes the formation of coarse grains and stable crystalline phases; however, insufficient release of internal stress may increase the long-term risk of delayed expansion. Forced-air cooling provides a moderate cooling rate, partially refines the particles, and results in an expansion behavior between that of naturally cooled and water-quenched slag. In contrast, water quenching induces rapid cooling, leading to the formation of more amorphous phases and a relatively loose microstructure. Although this process can enhance early hydration activity, the increased number of internal reaction channels may adversely affect the long-term volume stability of magnesium slag. The changes in the physicochemical properties of magnesium slag under different cooling regimes, namely ZLZ for naturally cooled magnesium slag, FLZ for forced-air-cooled magnesium slag, and SCZ for water-quenched magnesium slag, indicate that a shorter cooling time favors the formation of coarse magnesium slag particles with particle sizes greater than 75 μm. In addition, the specific surface area of magnesium slag decreases with increasing cooling rate from 1.8367 to 1.0571 m^2^/g. In terms of mineralogical composition, SCZ is mainly composed of β-C_2_S, whereas ZLZ is dominated by γ-C_2_S [31]. Zhu et al. [32] verified this view in their study of water-quenched magnesium slag, proving that the crystal structure of C_2_S is mainly affected by the cooling rate. Zhou et al. [33] also proved that the crystal structure of C_2_S can be regulated by the addition of metal oxides (BaO, B_2_O_3_, and P_2_O_5_) during magnesium slag production.

Regarding the volume expansion and long-term dimensional stability issues of magnesium slag-based cement materials, the Le Chatelier test is a classic and traditional method for detecting the expansion risks caused by free calcium oxide and free magnesium.

The Le Chatelier test follows standard industrial specifications. This method involves loading fresh slurry prepared from the material to be tested into standard Le Chatelier molds and curing them under specified temperature and humidity conditions. After the predetermined curing period, the expansion value of the sample is measured. The larger the measured expansion value, the higher the content of unstable oxides and the greater the risk of volume deformation. This method is simple to operate, has low equipment requirements, and is widely used in laboratories and on-site engineering for the rapid screening of raw material quality.

## 3. The Mechanism of the Volume Stability Influence of Magnesium Slag-Based Cementitious Materials

The chemical composition of magnesium slag is similar to that of Portland cement clinker, from which its potential to be used as a cementitious material is theoretically conferred [34]. However, a major challenge is faced by magnesium slag-based cementitious materials in practical applications, namely their poor volume stability. It is worth noting that significant volume expansion after hydration is exhibited by products such as f-CaO and f-MgO in magnesium slag [35]. In the field of cementitious materials, uncontrolled expansion has been proven to be harmful, while greater application value may be brought about by appropriate regulation of its expansion characteristics.

### 3.1. Hydration Expansion of Free Magnesium Oxide

Magnesium slag (MS) contains high content of f-MgO, which is recognized as the core factor leading to the long-term volume instability of MS-based binders. The hydration reaction of free magnesia follows the chemical equation below:

MgO + H_2_O → Mg(OH)_2_ (1)

This hydration reaction is a thermodynamically spontaneous exothermic reaction under normal temperature and curing conditions, with negative standard Gibbs free energy change (ΔG < 0) and negative standard enthalpy change (ΔH < 0). Two types of f-MgO are contained in magnesium slag: light-burned f-MgO and heavy-burned f-MgO [36]. Light-burned f-MgO is generated below 1200 °C and can react rapidly with water to cause volume expansion. Heavy-burned f-MgO is generated above 1450 °C and is characterized by low hydration activity and slow hydration over time. During the hydration process, magnesium hydroxide (Mg(OH)_2_) is generated by f-MgO, and significant volume expansion (approximately 118%) results from this reaction [37]. Unlike the rapid hydration of highly active calcium oxide (f-CaO), the hydration of f-MgO is a relatively slow process, by which occurrence may be exhibited only after the material has hardened or even been put into use [38]. Huge expansion stresses within the formed magnesium slag-based materials are induced by this lagging expansion, by which macroscopic cracking is caused and the long-term durability and structural integrity of the materials are seriously affected [39].

Kinetically, the hydration of f-MgO in the hardened cement matrix conforms to the diffusion-controlled reaction model. In the early stage, water molecules contact f-MgO particles and trigger rapid surface reactions. Soon afterwards, dense magnesium hydroxide (Mg(OH)_2_) hydration products wrap the residual unreacted f-MgO particles, forming a dense isolation layer. This layer blocks the diffusion channel of water molecules and sharply reduces the reaction rate.

The whole hydration process can be divided into three typical stages: the initial rapid reaction stage, the middle diffusion-limited slow reaction stage, and the long-term sustained hydration stage. Different from ordinary cement hydration, the slow hydration stage of f-MgO can last for several months or even years after the material hardens and is put into service. Garrault et al.’s research pointed out that the traditional view that the volume expansion caused by MgO expansion agents in cement-based materials is solely attributed to the formation of water–magnesite is insufficient. The expansion deformation mechanism is more complex [40]. Yang et al. [41]’s research also explored the influence of impurities in magnesium slag during the calcination process on the performance of the MgO expansion agent, further revealing its hydration characteristics. Such delayed hydration is the root cause of late expansion damage of MS-based materials.

Therefore, how the hydration activity and expansion effect of f-MgO can be effectively controlled is the core issue for the improvement of the volume stability of magnesium slag-based cementitious materials [42].

### 3.2. Hydration Expansion of Free Calcium Oxide

Besides f-MgO, f-CaO is another unstable component widely existing in magnesium slag. Its hydration behavior and expansion effect also pose adverse impacts on the volume stability of composite materials. The hydration reaction of free calcium oxide follows the following chemical equation:

CaO + H_2_O → Ca(OH)_2_ (2)

Similar to f-MgO hydration, the reaction of f-CaO with water is also a spontaneous exothermic process with negative ΔG and ΔH. Under the same curing environment, f-CaO has a higher thermodynamic hydration driving force than f-MgO, which fundamentally determines its faster reaction rate [43]. Li et al. [44] studied a simplified chemical–mechanical model of calcium oxide expansion. When calcium oxide comes into contact with water, it undergoes a rapid hydration reaction, generating Ca(OH)_2_ crystals whose volume is approximately twice that of the original calcium oxide particles. As a typical calcium–magnesium metallurgical solid waste, converter steel slag shares similar chemical compositions and unstable components with magnesium slag. Relevant thermodynamic studies have confirmed that the hydration reaction of f-CaO in various metallurgical slags is thermodynamically spontaneous across conventional service temperature ranges; once exposed to moisture, f-CaO will continuously undergo hydration and trigger irreversible volume expansion of slag-based building materials. Among all unstable components in calcium–magnesium slag systems, f-CaO is identified as the primary factor causing volume distortion, and its strong thermodynamic reaction tendency cannot be naturally eliminated during material hardening and long-term service [45].

**Figure 3:**
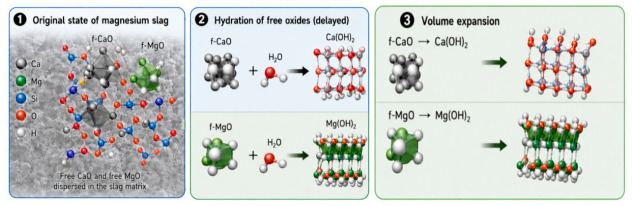
The hydration reaction and expansion mechanism of magnesium slag.

From the kinetic perspective, f-CaO hydration belongs to the surface reaction-controlled process. Most hydration reactions are completed in the early hardening stage (0–7 d), and the generated calcium hydroxide Ca(OH)_2_ product layer cannot effectively hinder the contact between water and residual f-CaO particles. Different from the long-term delayed hydration of f-MgO, f-CaO mainly causes expansion deformation in the early stage of material hardening. Dynamic experimental results on converter steel slag further verify the kinetic law of f-CaO in metallurgical slags. The hydration process of f-CaO is dominated by rapid surface reaction, and the generated Ca(OH)_2_ hydration products fail to form a dense isolation layer to block the diffusion of water molecules. Even if part of f-CaO is wrapped by hydration products, water can still contact unreacted f-CaO particles continuously. For this reason, the main hydration process of f-CaO is concentrated in the early curing period, and the reaction rate will not decrease significantly with the extension of time in the early stage. This kinetic feature leads to concentrated expansion stress in the early hardening stage of slag-based materials, which easily induces internal microcracks. The expansion mechanism diagram caused by two unstable components in the magnesium slag is shown in Figure 3.

## 4. Strategies for Improving the Volume Stability of Magnesium Slag-Based Cementitious Materials

The volume instability of magnesium slag-based cementitious materials mainly results from the delayed hydration expansion of free calcium oxide and free magnesium oxide [46]. This severely restricts their safe large-scale application in structural engineering [47]. To address these issues and ensure long-term stability, it is urgent to develop targeted control and improvement strategies. This section systematically summarizes some strategies for solving the volume stability of magnesium slag-based cementitious materials, and analyzes their mechanism and effect [48].

### 4.1. Modification of Magnesium Slag

The methods for modifying magnesium slag include physical modification and chemical modification [49]. Physical modification reduces the particle size of magnesium slag and increases the specific surface area through methods such as grinding. This method is simple but its effect is not as good as that of chemical modification. Therefore, this section mainly focuses on the chemical modification of magnesium slag and explores methods to improve the volume stability of magnesium slag-based materials through chemical modification means [50].

The common methods for chemical modification of magnesium slag include alkaline activation, acid treatment, additive modification, etc. Compared with the unmodified magnesium slag (UMS), the modified magnesium slag has excellent properties. Li et al. [51] conducted a systematic study on the modification and pretreatment of magnesium slag using formic acid, acetic acid, and phosphoric acid. The research showed that the acid modification process would have an etching effect on the magnesium slag particles, causing the surface to form a rough and porous microstructure, significantly reducing the particle size and greatly increasing the specific surface area. Among them, the acetic acid modification (AMSC) increased the specific surface area of the magnesium slag by 325%, the formic acid modification (FMSC) by 275%, and the phosphoric acid modification (PMSC) by 225%. Compared with the control concrete mixed with unmodified magnesium slag, after adding the modified magnesium slag (formic acid- or acetic acid-modified) to the cement, the 28-day compressive strength increased by 17.51% and 22.56% respectively. Moreover, the pore structure of the composite cementitious system was significantly optimized, the water process was accelerated, and the slurry was denser, confirming that the acid modification pretreatment of magnesium slag is beneficial.

**Figure 4:**
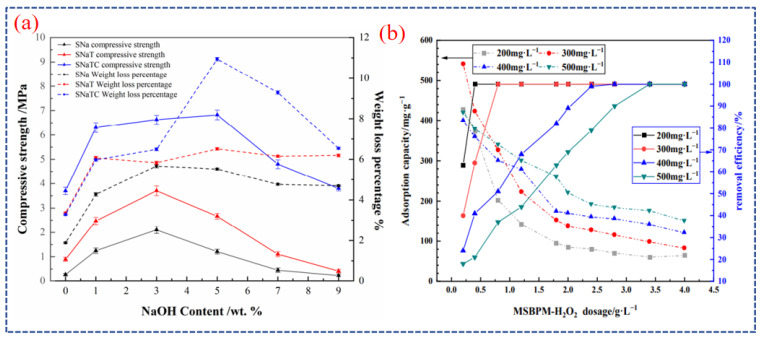
(**a**) Compressive strength of specimens with different sodium hydroxide contents, adapted from Ref. [52]; (**b**) Pb^2+^ adsorption capacity under hydrogen peroxide treatment, adapted from Ref. [53].

Alkali activation modification of magnesium slag is also a good method. Zhu et al. [52] systematically studied the processes of the alkaline activation and carbonation modification of magnesium slag using sodium hydroxide (NaOH) as an activator. The research results showed that alkaline activation increased the pH value of the reaction system, promoted the dissolution of calcium and silicon ions in the magnesium slag, and facilitated the formation of hydration products including Ca(OH)_2_ and C-S-H. After being cured for 24 h and then carbonized for 1 h, the maximum compressive strength of the modified magnesium slag composite material reached 6.83 megapascals, and the carbon dioxide sequestration capacity reached as high as 4.74% (by mass percentage), indicating that alkaline activation and carbonation modification significantly enhanced the mechanical properties and carbon fixation capacity of the magnesium slag (Figure 4a). Lu et al. [53] successfully synthesized magnesium slag-based porous materials (MSBPMs) through alkaline activation combined with foaming technology (using hydrogen peroxide and aluminum powder as foaming agents). The research results showed that the alkaline activation and foaming regulation could enable the formation of abundant pore structures within the magnesium slag, and the optimized material had a compressive strength of 8.46 MPa and a Pb^2+^ adsorption capacity of 396.11 mg·g^−1^. This alkaline activation foaming modification not only enhanced the structural stability and mechanical properties of the magnesium slag, but also endowed it with efficient heavy metal adsorption capacity (Figure 4b).

In addition to the acid–base treatment of magnesium slag, adding stabilizers during the magnesium smelting process to modify the composition of the magnesium slag is also a popular method. Xie et al. [54] conducted a systematic study on the source modification of magnesium slag. The research results showed that source-additive modification could effectively inhibit the volume expansion and pulverization defects of magnesium slag during cooling, stabilize the highly active β-C_2_S mineral phase, and significantly improve the grindability and hydration activity. The compressive strength of the magnesium slag-based material with 20% modified content increased by approximately 3 MPa after 28 days, and its early hydration heat release was significantly higher, indicating a strong hydration composite effect between the MMS and clinker. Ruan et al. [55] also carried out modification treatment on the source of magnesium slag. The research results showed that the modified magnesium slag (MMS) maintained a stable block-like shape, with smaller particle sizes and better grindability. Microscopic analysis revealed that the slurry based on MMS generated more C–S–H and Ca(OH)_2_ and a small amount of ettlingerite minerals, enhancing the strength and forming a microstructure with a denser structure, fewer pores, and stronger interfacial bonding force.

All chemical and physical modification techniques can mitigate the early volume expansion of magnesium slag, but they have inevitable drawbacks in application. Acid-, alkali- and stabilizer-based chemical methods show desirable modification effects, while they are restricted by unstable treatment performance, extra costs, environmental pollution risks and potential deterioration in long-term durability. Physical modification is environmentally benign but can only serve as an auxiliary measure, since it cannot fundamentally eliminate expansive components inside the slag. Currently, most relevant studies concentrate on short-term performance under laboratory conditions, and there is a lack of in-depth exploration on long-term stability and adaptability in complex service environments, which restricts the further popularization of these modification technologies [56].

### 4.2. The Synergistic Effect of Magnesium Slag with Other Solid Waste-Based Materials

Magnesium slag (MS) exhibits remarkable synergistic effects when combined with other solid waste-based materials such as granulated blast furnace slag (GGBS), fly ash, and steel slag in cementitious systems, which can effectively compensate for the limitations of a single MS application; further optimize the hydration process, mechanical properties, volume stability, and durability of composite materials; and provide a feasible way for the collaborative resource utilization of various industrial solid wastes.

#### 4.2.1. The Synergistic Effect of Magnesium Slag and Ground Granulated Blast Furnace Slag

Some scholars have already conducted research on the collaborative utilization of magnesium slag and blast furnace slag. Zhu et al. [32] systematically studied the reinforcing effect of granulated blast furnace slag (GGBS) on the cement-based solidification and backfilling material of modified magnesium slag. The research results showed that GGBS could significantly shorten the induction period and acceleration period of the hydration reaction, advance the second exothermic peak, and increase the total hydration heat release by up to 62%. With the increase in the content of GGBS, the uniaxial compressive strength at 3 days and 7 days significantly increased, and the strength at 28 days and 56 days also improved significantly. Compared with the control group, adding 1% wt% of GBFS increased the 28-day strength by 29.7% and the 56-day strength by 26.1%. At the same time, more hydration products, such as C-S-H and ettringite, were generated, and the microstructure became denser, indicating that GBFS had a significant reinforcing effect on the magnesium slag-based backfill material.

Zhuang et al. [57] systematically revealed the synergistic mechanism of MS and granulated GGBS in the alkaline activation system. The study showed that there is a two-stage synergistic reaction between MS and GGBS: the initial reaction of GGBS promotes the hydration of MS, while the products of MS further accelerate the reaction of GGBS, and at the same time have good strength performance, confirming that there is a significant synergistic effect between magnesium slag and blast furnace slag.

**Figure 5:**
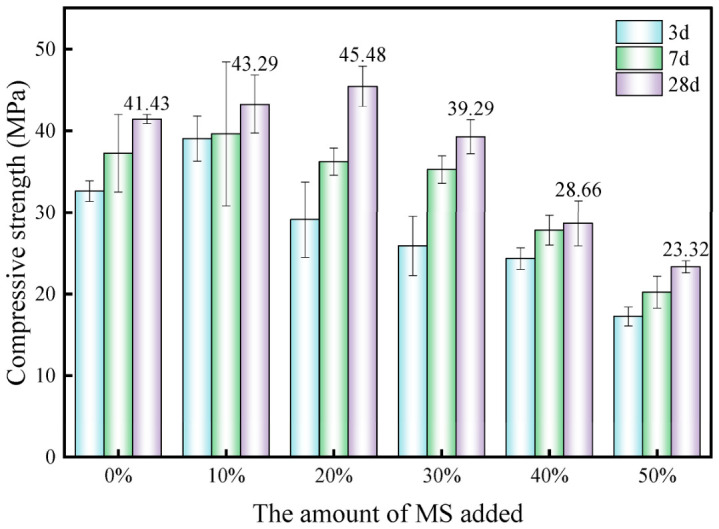
Compressive strengths of samples with different replacement amounts of MS, adapted from Ref. [30].

Lu et al. [30] systematically compared the structural characteristics and gelation properties of MS and GGBS. The results showed that under alkaline activation, the dissolution amounts of silicon and aluminum in granulated blast furnace slag were much higher than those in magnesium slag. When used as a cement substitute, a 20% substitution amount of magnesium slag could achieve the highest 28-day compressive strength of 45.48 MPa, while granulated blast furnace slag could still maintain higher strength at a 50% substitution amount (Figure 5). Although the activity of magnesium slag is lower than that of granulated blast furnace slag, both can be used as supplementary gelling materials, providing a basis for their collaborative application.

Yang et al. [58] systematically investigated the effects of MS and GGBS as partial cement substitutes on the performance of cement paste ending rock backfill materials. The results showed that the strength of all backfill specimens increased with the increase in curing time, and they also exhibited good elastic modulus and strength reduction performance. These findings confirmed that GGBS and MS can be used synergistically for mine backfilling.

The research conducted by the aforementioned scholars indicates that the combined utilization of magnesium slag and granulated blast furnace slag effectively overcomes the shortcomings of magnesium slag alone, such as slow early hydration and insufficient gelation activity. Through complementary activities and bidirectional stimulating effects, blast furnace slag can significantly accelerate the hydration process of the system, increase the heat of hydration, greatly enhance the early and long-term mechanical strength, and promote the formation of more hydration products such as C-S-H and calcium aluminosilicates, resulting in a dense microstructure. In the alkali activation system, the two substances exhibit a two-level synergistic reaction characteristic, mutually promoting hydration, optimizing the composition and microstructure of the products, and simultaneously improving the volume stability and reducing the risk of shrinkage cracking. Although the compound system of magnesium slag and GGBS shows good comprehensive performance, the mixing ratio has strict restrictions. Excess magnesium slag will make the overall expansion go out of control; excessive GGBS will increase the material cost. In addition, GGBS is sensitive to curing temperature and water–binder ratio, so the construction adaptability of the composite material is poor. Current research mostly focuses on mechanical properties and early expansion, while the co-evolution rule of the carbonation shrinkage and chloride resistance of the binary system in long-term service is still lacking in-depth discussion.

#### 4.2.2. The Synergistic Effect of Magnesium Slag and Steel Slag

Magnesium slag and steel slag are both typical calcium–magnesium-type metallurgical solid wastes. They have similar chemical compositions and mineral compositions. Their combined application can effectively overcome the shortcomings of single utilization.

**Figure 6:**
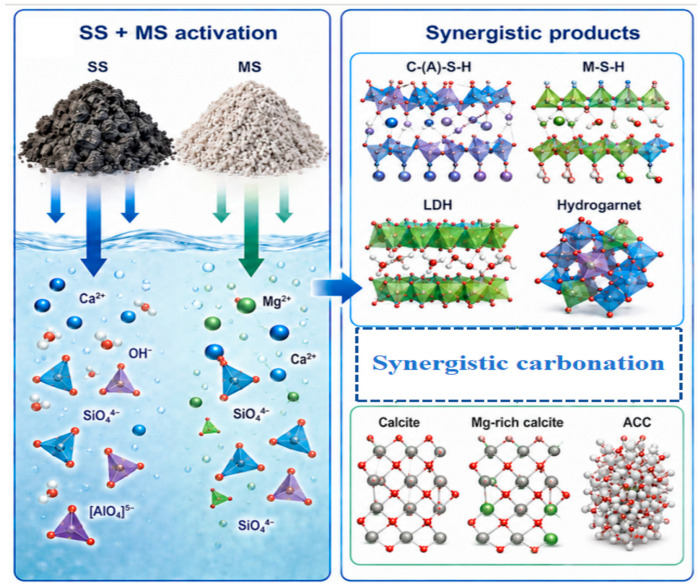
The collaborative reaction mechanism of MS-SS.

Yuan et al. [59] conducted a systematic study on the mechanism of the synergistic carbonation reaction between magnesium slag and steel slag under suspended conditions. The research showed that this synergistic system could significantly enhance the carbon dioxide fixation efficiency of magnesium slag and steel slag, with a carbon dioxide adsorption rate of up to 5% within 5 min and a carbonation degree of 7%. In the case of low steel slag content, magnesium ions (Mg^2+^) replaced calcium ions (Ca^2+^) to enter calcite and form magnesium-rich calcite, and as the proportion of steel slag increased, the composite system produced more calcite and amorphous calcium carbonate (ACC). Compared with the single-carbonation systems of magnesium slag or steel slag, the synergistic carbonation of magnesium slag and steel slag significantly promoted the precipitation of calcium ions (Ca^2+^), accelerated the formation of carbonate products, and confirmed that the synergistic utilization of magnesium slag and steel slag is conducive to improving the carbonation fixation efficiency and improving the microstructure of the material (Figure 6).

Nong et al. [60] systematically investigated the reaction behavior, microstructure evolution and performance development of alkali-activated magnesium slag and steel slag materials. The results indicated that steel slag exhibited higher reactivity in alkaline environments, forming C-A-S-H, LDH, and hydrogarnet, while magnesium slag needed higher alkalinity to activate the formation of C-A-S-H and M-S-H. The synergistic system of magnesium slag and steel slag realized complementary advantages, the reasonable ratio significantly increased the compressive strength of the consolidated body, and the 28-day strength could reach 8.0–21.8 MPa. Both of them could effectively immobilize heavy metals, and the composite system reduced the usage of alkali excitant and improved the economic and environmental benefits. In addition, the synergistic effect refined the pore structure, reduced the total porosity and inhibited the microcracks caused by excessive expansion of magnesium slag, confirming that the synergistic use of magnesium slag and steel slag is beneficial to the performance promotion of alkali-activated cementitious materials.

Zhang et al. [61] systematically developed magnesium slag–steel slag-based composite cementitious materials and applied them to mine backfill. The study demonstrated that the optimal ratio of the synergistic cementitious material was 30% magnesium slag, 15% steel slag, 6% desulfurization gypsum and 49% blast furnace slag. Magnesium slag compensated for the insufficient early hydration of steel slag and desulfurization gypsum, prolonged the hydration reaction time and maintained a high reaction rate in the later stage. The hydration products such as ettringite and C-S-H were interwoven to form a dense microstructure, and the 28-day compressive strength met the mine backfill index. Under the condition of a slurry concentration of 78% and binder–aggregate ratio of 1:5, the filling cost was reduced by 42.19% compared with traditional cement materials. Moreover, the synergistic system improved the rheological properties of the filling slurry, reduced the pipeline conveying resistance, and confirmed that the synergistic utilization of magnesium slag and steel slag is conducive to preparing low-cost and high-performance mine backfill materials.

In conclusion, the combination of magnesium slag and steel slag can enhance the carbon sequestration efficiency, optimize the microstructure, prevent expansion cracking, improve the stability of magnesium slag-based materials, and achieve efficient utilization of both solid wastes. However, both magnesium slag and steel slag contain expansive oxides, so the composite system of these materials has the risk of cumulative volume deformation. Although carbonation activation can alleviate the expansion to some extent, it is difficult to control the degree of the carbonation reaction in actual engineering. Insufficient carbonation cannot suppress the expansion, while excessive carbonation will lead to severe contraction.

#### 4.2.3. The Synergistic Effect of Magnesium Slag and Fly Ash

Magnesium slag (MS) and fly ash (FA) are both typical industrial solid wastes with potential for volcanic ash activity. Their combined application in cement-based systems can effectively overcome the deficiency of the low hydration activity of magnesium slag alone, optimize the hydration process, and improve mechanical properties and volume stability. This section summarizes the collaborative utilization methods of magnesium slag and fly ash, and provides a way of thinking regarding the collaborative resource utilization of these two types of solid wastes.

Yang et al. [62] systematically investigated the mechanical properties, pore characteristics and microstructure of modified magnesium slag cement-based coal-based solid waste backfill materials that were added with fly ash and treated at different curing temperatures. The study showed that fly ash could provide additional active particles to participate in the hydration reaction and promote the formation of flocculent C-S-H and needle-like Ettringite calcium aluminosilicate, thereby significantly optimizing the pore structure and reducing the porosity. With the addition of fly ash increasing from 0% to 40%, the 28-day uniaxial compressive strength increased from 2.503 megapascals to 6.063 megapascals, and a good linear correlation was observed between the fractal dimension and the strength. This confirmed that the synergistic use of magnesium slag and fly ash is beneficial to the improvement in the performance of full solid waste backfill materials.

**Figure 7:**
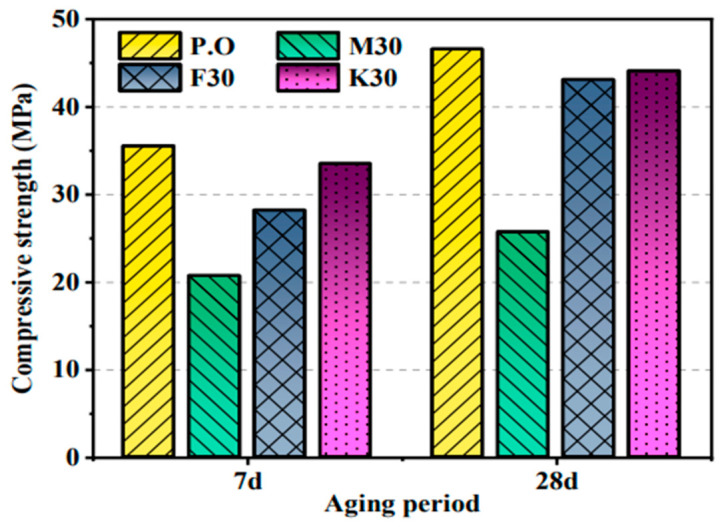
The 7- and 28-day compressive strength, adapted from Ref. [63].

Wei et al. [63] systematically explored the feasibility of magnesium slag, fly ash and metakaolin in replacing part of cement as cementitious materials. The research showed that the 28-day activity index of single magnesium slag was only 55.15%, while the synergistic system with fly ash and metakaolin exhibited excellent performance. The optimal ratio of magnesium slag:fly ash:metakaolin:cement was 10:10:10:70, and the 28-day compressive strength reached 38.5 MPa, which was 144.2% higher than that of solely magnesium slag mortar (Figure 7). Fly ash compensated for the later strength deficiency of magnesium slag, and magnesium slag alleviated the drying shrinkage of cement mortar, while the composite system refined the pore size and improved the compactness. In addition, the heavy metal leaching risk was low, confirming that the synergistic use of magnesium slag and fly ash is beneficial to the development of green and low-carbon cementitious materials.

Fang et al. [64] systematically analyzed the hydration behavior, rheological control and strength performance of the magnesium slag–fly ash–cement ternary cementitious material in the fluidizable solidified soil. The research results showed that magnesium slag provided active CaO and C_2_S, which significantly increased the release of hydration heat and promoted the formation of C-S-H gel and Ca(OH)_2_. Compared with the binary system of fly ash–cement, the 28-day compressive strength of the ternary system in the paste increased by up to 22.7%, while the microstructure became denser and harmful pores were reduced. 

Liu et al. [65] systematically studied the synergistic disposal effect of magnesium slag and high-calcium fly ash as cementitious materials for mine backfilling. The research results showed that as the addition amount of fly ash increased from 0% to 40%, the unconfined compressive strength at 28 days increased from 2.607 megapascals to 7.491 megapascals, and the early strength development rate significantly accelerated. The hydration process of magnesium slag releases Ca(OH)_2_, activating the pozzolanic reaction of fly ash, generating a large amount of C-S-H, forming a dense microstructure, and enhancing the structural integrity.

Gao et al. [66] compared the strength and hydration mechanisms of binary and ternary mixed cement mortars containing magnesite slag and fly ash. The study revealed that the optimal ratio of the magnesite slag–fly ash–cement ternary system was 10:10:80. The synergistic reaction between magnesite slag and fly ash promoted the consumption of Ca(OH)_2_, produced more hydration products, and improved the compactness of the microstructure. Moreover, the synergistic system reduced the amount of cement used and carbon emissions, confirming that the synergistic use of magnesite slag and fly ash is beneficial for the preparation of environmentally friendly and high-performance cement-based materials.

Nevertheless, it should be noted that fly ash is a by-product of coal-fired power plants. With the global adjustment of the energy structure and the gradual reduction in thermoelectric facilities in many regions, the supply of fly ash will face continuous shrinkages. Accordingly, the collaborative utilization of magnesium slag and fly ash is more suitable for short- and medium-term applications, and cannot fully meet the demands of long-term industrial development. It is urgent to explore alternative solid waste resources to form more sustainable synergistic systems with magnesium slag.

The collaborative utilization of magnesium slag with three typical industrial solid wastes, namely blast furnace slag, steel slag, and fly ash, effectively addresses key issues such as low hydration activity, poor volume stability, and easy expansion and cracking of solely magnesium slag-based cementitious materials. Currently, new solid waste materials need to be developed for collaborative utilization with magnesium slag to enhance its volume stability. The treatment methods for magnesium slag mentioned in this article are shown in Table 1.

### 4.3. Comprehensive Comparison and Overall Evaluation

To sum up, various modification and collaborative utilization technologies have their own applicable scenarios and inherent defects. Chemical modification works well in expansion control, but is restricted by cost, environmental pollution and long-term durability risks. Physical modification is green and safe, but the regulation effect is limited and cannot eradicate volume instability. Multi-solid waste compounding is the most promising technical route for industrial application, but it is faced with unclear interaction mechanisms, strict proportion limitation and insufficient long-term service data.

At present, the existing research still has two common deficiencies: first, most studies focus on a single performance index under standard laboratory conditions, and lack systematic evaluation on multi-index coupling performance such as long-term volume change, carbonation resistance and chloride penetration; second, the research on mechanisms stays at the macroscopic phenomenon analysis, and the micro-scale evolution of unstable phases under different modification means is not deeply revealed. In the future, it is necessary to combine multi-scale characterization and long-term aging tests to break through the existing technical bottlenecks.

## 5. Summary and Prospect

### 5.1. Summary

This review has systematically elucidated that the volumetric instability of magnesium slag-based materials is principally governed by the delayed hydration expansion of f-CaO and f-MgO, coupled with deleterious phase transformations. To mitigate these detrimental effects, the efficacy of intrinsic slag modification and synergistic blending with supplementary cementitious materials, including granulated blast furnace slag, steel slag, and fly ash, has been critically validated. These strategies have been demonstrated to substantially enhance dimensional stability, thereby unlocking the latent potential of magnesium slag for sustainable construction applications.

### 5.2. Prospect

The resource utilization of magnesium slag within construction materials has been recognized as a pivotal pathway toward sustainable development and carbon emission reduction. Notwithstanding the comprehensive elaboration of volume stability mechanisms and mitigation strategies presented herein, several formidable challenges and expansive research prospects have been identified for future exploration:

(1) The scope of synergistic solid waste utilization with magnesium slag must be substantially broadened. While the interactions with granulated blast furnace slag, steel slag, and fly ash have been critically examined, numerous other underutilized streams remain unexplored. These include red mud, phosphogypsum, coal gangue, and construction and demolition wastes. Exploring the synergistic mechanisms between various solid wastes and magnesium slag helps develop high-performance and low-carbon composite cementitious materials. This approach overcomes the limitations of single-waste utilization and broadens the engineering applications of magnesium slag.

(2) The long-term environmental safety and ecological risks associated with modified magnesium slag require in-depth study. Although the cementation performance has significantly improved after modification, the long-term environmental durability of the modified magnesium slag materials under actual usage conditions still needs to be carefully evaluated. Therefore, the development of new and environmentally friendly modification methods has been listed as an urgent research priority.

(3) The heavy metal immobilization capacity of magnesium slag has been investigated only to a conspicuously limited extent. The intrinsic determinants governing heavy metal speciation and fixation efficacy have yet to be elucidated mechanistically. Future endeavors should be directed toward a systematic exploration of process parameters and mineralogical evolution on stabilization performance.

## Figures and Tables

**Table 1:** The treatment methods and characteristics of magnesium slag.

Category	Treatment/Mixing Mode	Main Physicochemical Properties	Activity
Unmodified MS	Natural cooling	Main components: CaO: approximately 55%; SiO_2_: approximately 30%. True density 2.86–2.92 g/cm^3^; specific surface area 1.06–1.84 m^2^/g (decreases with higher cooling rate)	Low early activity, slow water absorption [30,31]
	Forced air cooling		
	Water-quenched cooling		
Modified MS	Acid modification	Chemical composition unchanged; specific surface area greatly increased	Compressive strength improved [51]
	Alkali activation	Chemical composition unchanged; specific surface area increased	Compressive strength improved [53]
	Source addition of stabilizers	Particle size reduced; grindability enhanced	Higher hydration activity [54]
Composite systems	MS + GGBS	-	28-day compressive strength enhanced [57,60,64]
	MS + SS	-	
	MS + FA	-	

## Data Availability

No new data were created or analyzed in this study. Data sharing is not applicable to this article.
